# Spreading Dynamics in Complex Networks

**DOI:** 10.3390/e28101149

**Published:** 2026-10-20

**Authors:** Lazaros K. Gallos

**Affiliations:** DIMACS, Rutgers, The State University of New Jersey, Piscataway, NJ 08854, USA; lgallos@gmail.com

The complexity of systems in the physical, social, biological, or technological world arises from the need for the fundamental system units (network nodes) to interact with one another (represented by network links), enabling the system to perform a variety of functions [1]. The aggregate of all interactions among the nodes can be viewed as a complex network that may also evolve over time [2,3]. This network forms the substrate that facilitates the exchange of ‘information’ between interacting nodes and, eventually, throughout the system [4,5]. This information can correspond to literal data, biological pathogens, behavioral traits, physical objects, or any other type of information that is transferred through the network [6]. The versatility of this framework becomes obvious when we assign concrete meaning to nodes, links, and information. For example, in a contact social network setting, we can connect people (nodes) who come into physical proximity on a given day (links) [7]. Individuals who are infected with a pathogen (‘information’) can potentially spread it to their network contacts, and this setup allows us to monitor how the virus spreads within this system [8]. Similarly, a technological network, such as the Internet of Things, can connect devices (nodes) via communication links, either wired or wireless [9]. The spreading paradigm can be used in this case to assess the resilience of such a network by examining how attacks and/or failures may propagate throughout the system [10].

Spreading dynamics in complex networks was traditionally examined using classical compartmentalized epidemiological models, such as SIR or SIS [11], which assume homogeneous mixing among populations. With the explosion of data availability in recent decades, network science has led a paradigm shift by demonstrating how varying architectures, including combinations of long-tail degree distributions [12,13], small-world features, modularity, spatial embedding, and others, fundamentally modify the simplistic pictures of homogeneous compartmental models. While the core mechanics of these classical frameworks remain central to the study of spreading, their integration with data-driven topologies has vastly improved our understanding of, among other things, spreading pathways, epidemic thresholds, and the conditions for the emergence of cascades or phase transitions in multiple real-world systems [14].

This Special Issue, titled “Spreading Dynamics in Complex Networks”, was purposefully designed to include a broad array of topics. The variety of papers in this Special Issue reflects the wide range of problems, methods, and applications across multiple disciplines that fall under the umbrella term of spreading in complex systems. Many papers in this Special Issue focus on social networks and information dynamics [15,16,17,18,19,20], theoretical or data-driven, and some specifically address epidemic modeling in human contact networks [19,20]. Applications are represented in a variety of settings, including the Internet of Things [9], or linguistic effects on stock market volatility [21], while theoretical advances of the forest-fire model on Bethe lattices highlight the importance of clusters in this model [22]. The proliferation of widely available artificial intelligence tools carries the promise of revolutionizing what is possible in scientific research, and of course this includes complex systems research. As such, two papers in this Special Issue combine advanced graph machine learning with network analysis [23,24], highlighting the promise of new and exciting AI-focused research.

The study of spreading in networks has a rich history and a promising future, as a topic that remains at the forefront of interdisciplinary research. Theoretical advancements can make a real difference when dealing with extremely complex situations, such as those encountered in real-world problems. Typical applications range from world pandemics or local virus spreading to information spreading over social networks and to the vulnerability of technological or financial networks.

Consequently, the field is expected to remain very active in the foreseeable future, driven by the ongoing goal of incorporating the increasingly complex characteristics of real-world situations. In this context, the list below summarizes a few of the current research directions and challenges.

(a) Multi-layer structures. Most real-world networks interact with other networks [25]. For example, transportation networks continuously exchange passengers, as people transfer from cars to trains or planes, so that road traffic networks are mutually dependent on train and plane networks. Similarly, individuals participate in multiple social networks, enabling the immediate transfer of information between these networks [26]. Studying each network independently of the others cannot capture how each system works, as it ignores the continuous changes arising from inter-network interactions [27]. This is a flourishing field, and it has generated a plethora of new results, even in fields such as Physics [28], while highlighting new phenomena in spreading.
(b) *Higher-order structures.* Interactions in real networks frequently involve groups rather than pairs of nodes [29]. The study of simplicial complexes and hypergraphs [30] enables the study of group interactions as the fundamental network unit, rather than pairwise interactions alone [31].
(c) *Temporal evolution.* Systems evolve dynamically over time, and in many cases, the rate of these changes is comparable to or faster than the spreading rates [32]. The interplay between spreading and network topology is an important aspect, as one directly affects the other [33,34]. For example, the goal of the quarantine measures during the first stages of the COVID-19 pandemic was to drastically change the topology of the human contact network and make the network sparser [35]. The resulting changes had to be continuously incorporated into the models predicting the spreading patterns.
(d) *Information-theoretic metrics.* The use of concepts like transfer entropy, mutual information [36], and structural entropy, rooted in information theory [37], allows us to study spreading in networks through a different perspective [38,39], and has been used to quantify directional information flow [40], identify hidden correlation structures [41], and evaluate the system robustness against targeted attacks or random node failures. The importance of information theory in current communications and the rapid development of the field suggest that similar approaches may offer novel ways of studying spreading dynamics [42].
(e) *Scaling to larger systems and uncertainty.* Many of the methods in the field require computationally intensive algorithms. Running those algorithms in multi-layer, higher-order, or evolving networks while scaling to massive, billion-node empirical datasets represents a significant challenge [43]. Another challenge is addressing the significant uncertainty in real-world data—ideally in real time—especially data describing human interactions and behavioral responses. The feedback between received (or perceived) information and an individual’s response to it can significantly influence the evolution of spreading [44]. The design of control strategies becomes an even greater challenge when these strategies must account for information uncertainty in a very large system [45].
(f) *Artificial intelligence.* Just like in every aspect of science, AI is expected to have a large impact on understanding spreading in networks. Advanced graph neural networks and machine learning models carry the promise of simulating and predicting epidemic phenomena with unprecedented precision across large-scale topological structures, potentially addressing many of the challenges described above within an integrated framework [46]. Reinforcement learning techniques could also greatly improve the design of intervention strategies and suggest efficient ways to prevent systemic failures [47]. However, there is always the risk of black-box solutions, and researchers should be very skeptical of adopting any AI-generated findings if they cannot understand the basic ‘science’ behind such results, or the limitations and the scope to which these findings may apply.

Our understanding of spreading dynamics has come a long way since the compartmental models were introduced in the early 20th century [11]. As research continues to expand the boundaries of what can be predicted and controlled in an increasingly complex world, these insights will remain as some of the important contributions of science to modern society, safeguarding public health, protecting critical technological infrastructures, and strengthening social systems.
